# Binaural Fusion and Listening Effort in Children Who Use Bilateral Cochlear Implants: A Psychoacoustic and Pupillometric Study

**DOI:** 10.1371/journal.pone.0117611

**Published:** 2015-02-10

**Authors:** Morrison M. Steel, Blake C. Papsin, Karen A. Gordon

**Affiliations:** 1 College of Medicine, Central Michigan University, Mount Pleasant, MI, United States of America; 2 Institute of Medical Science, University of Toronto, Toronto, Ontario, Canada; 3 Research Institute, The Hospital for Sick Children, Toronto, Ontario, Canada; 4 Department of Otolaryngology-Head and Neck Surgery, University of Toronto, Toronto, Ontario, Canada; 5 Department of Otolaryngology, The Hospital for Sick Children, Toronto, Ontario, Canada; 6 Department of Communication Disorders, The Hospital for Sick Children, Toronto, Ontario, Canada; Texas Christian University, UNITED STATES

## Abstract

Bilateral cochlear implants aim to provide hearing to both ears for children who are deaf and promote binaural/spatial hearing. Benefits are limited by mismatched devices and unilaterally-driven development which could compromise the normal integration of left and right ear input. We thus asked whether children hear a fused image (ie. 1 vs 2 sounds) from their bilateral implants and if this “binaural fusion” reduces listening effort. Binaural fusion was assessed by asking 25 deaf children with cochlear implants and 24 peers with normal hearing whether they heard one or two sounds when listening to bilaterally presented acoustic click-trains/electric pulses (250 Hz trains of 36 ms presented at 1 Hz). Reaction times and pupillary changes were recorded simultaneously to measure listening effort. Bilaterally implanted children heard one image of bilateral input less frequently than normal hearing peers, particularly when intensity levels on each side were balanced. Binaural fusion declined as brainstem asymmetries increased and age at implantation decreased. Children implanted later had access to acoustic input prior to implantation due to progressive deterioration of hearing. Increases in both pupil diameter and reaction time occurred as perception of binaural fusion decreased. Results indicate that, without binaural level cues, children have difficulty fusing input from their bilateral implants to perceive one sound which costs them increased listening effort. Brainstem asymmetries exacerbate this issue. By contrast, later implantation, reflecting longer access to bilateral acoustic hearing, may have supported development of auditory pathways underlying binaural fusion. Improved integration of bilateral cochlear implant signals for children is required to improve their binaural hearing.

## Introduction

Unilateral implant use causes abnormal reorganization of the auditory pathway at the level of the brainstem [1–4] and the cortex [5–7]. Bilateral cochlear implants (CIs) have been provided to children to promote binaural hearing [8–16] and ease the increased effort required for listening demonstrated by unilaterally implanted children [17,18]. Unfortunately, many differences between children using bilateral CIs and their normal hearing (NH) peers remain, such as greater variability in responses and increased reliance on CI-1 (first implanted CI) and interaural level [10,13,15,16]. Asymmetric development, poor neural survival, electrical stimulation, and mismatched places of stimulation could impact the ability of children who are deaf to perceptually integrate, or fuse, input delivered by bilateral implants and thereby impair binaural hearing [2,5,6,19–23]. Children with bilateral CIs may thus have limited success with their devices if binaural fusion does not occur [24]. The recent finding that sequentially implanted children reported hearing from both devices simultaneously rather than one image [15] most clearly suggests that binaural fusion is impaired. It remains unclear whether bilaterally implanted children have access to accurate binaural cues or integrate these cues similarly to children with intact NH. Therefore, we aimed to measure binaural fusion in children with bilateral CIs.

### Binaural Fusion is Affected More by Interaural Timing than Level Differences in Normal Hearing Listeners

Binaural fusion refers to the subjective perception of 1 versus 2 sounds, when presented with a sound stimulus in each ear, while binaural integration/interaction refers to objective neural responses. When signals have interaural/implant level or time differences (ILDs or ITDs) equal to zero, binaural sounds are perceived as coming from the center of the head (midline) in listeners with intact NH. When ILDs are increased, the sound is heard on one side of the head (ie. lateralized) [25]. When the ILD exceeds 15–20 dB, binaural input is perceived as a single fused monaural image on an extreme side of the head. Large ILDs do not interfere with fusion, or result in the perception of two distinct auditory images. ITDs are generally the dominant cue for localization in normal listeners [26–28]. In the horizontal plane, sound travels between the ears within a range of approximately ±0.7 ms ITDs for anechoic free sound field localization and ±1.0 ms ITDs for earphone-mediated click lateralization [25]. As click ITDs increase beyond this range, the position of the auditory image remains on an extreme side of the head, but two distinct sounds can be heard. This phenomenon has been referred to as the end point of lateralization [29].

### Integration of Binaural Input in the Brainstem and Cortex is likely Important for Fusion

The integration of binaural input via coincident counters in the superior olivary complex (SOC) [30] and/or interaural cross-correlation at multiple levels in the system [31] may underlie the perception of a fused auditory image. The lateral and medial nuclei of the mammalian SOC (LSO and MSO) are specialized for ILD and ITD processing, respectively [32,33]. Regardless of the specific mechanism of ITD coding in mammals [34–36], large mismatches in interaural place of stimulation reduce binaural fusion [24,37]. In general, similar enough areas in the two cochleae must be stimulated [37,38] at similar times [30,33] in order for binaural integration to be possible at higher levels. Physiological integration can be represented at the level of the auditory brainstem by large binaural difference response amplitudes when interaural mismatches are minimal [4,39]. The binaural difference response is an electrophysiological measure that is recognized in animals [40], normal listeners [39,41] and CI users [4]. This difference measure is calculated by subtracting the amplitude of binaurally evoked potentials from the sum of those evoked monaurally [42] and is thought to reflect inhibition in the SOC [43,44]. The β component (baseline-to-peak amplitude) of the difference waveform is undetectable for ITDs larger than ~1 ms [25], which is consistent with data from behavioral tasks assessing binaural fusion [45].

Activity in the cortex is also believed to reflect integration [46]. When cortical activity was recorded with electroencephalography during a passive oddball task, the mismatch negativity waveform was only non-significant for stimuli presented dichotically and with small interaural frequency differences. This finding is consistent with single-unit recordings from the cat auditory cortex which demonstrate that activity remains constant in the presence of dichotic stimuli registered as a single fused image [47]. Matched dichotic stimuli, or those with small interaural differences, thus appear to be integrated throughout the ascending auditory pathway.

### Children with Normal Hearing Perceive Small Interaural Timing Differences as Fused Auditory Images

Behaviorally, binaural integration has been measured as a form of auditory temporal resolution or, more precisely, auditory fusion. Temporal resolution underlies many auditory and auditory-language processes and has been quantified with the use of various psychophysical measures [45]. As it stands, the clinical tests most commonly used to assess temporal resolution are the Auditory Fusion Test-Revised, the Random Gap Detection Test for Tones/Clicks, the Gaps-In-Noise for the Right/Left ear, and the Binaural Fusion Test. Because dichotic stimuli have been used to assess binaural integration objectively [39,46,47], psychophysical tests that use dichotic stimuli to assess fusion (eg. Binaural Fusion Test) were considered to be most relevant for the present study. For dichotic stimuli, interaural stimulus intervals greater than 1 ms have been found to decrease fusion in NH children [45]. However, binaural fusion has not yet been measured in children using bilateral CIs and is likely to be disrupted in these individuals for several reasons.

### Unilateral CI Use Promotes Auditory Perception in Deaf Children

While only a small subset of auditory neurons are needed to facilitate auditory perception, sensorineural hearing loss causes demyelination, decreased cell size, slower neural conduction, and reduced sensitivity to binaural cues [48–53]. Early auditory experience is critical for normal language acquisition, speech perception, sound localization, and central auditory development [54], which can be compromised by severe-to-profound deafness in childhood [51,55]. Longer periods of deafness leave auditory association cortices vulnerable to becoming decoupled from primary auditory cortex [56] and taken-over by visual [57,58] and somatosensory areas [59], which may limit the potential for later reactivation with CI stimulation. CIs are the most successful neural prosthetic device and bypass damaged hair cells to directly stimulate the spiral ganglion with customized electrical pulses [22]. CI use in children improves speech recognition, production, and intelligibility [60–62] and promotes emotion perception [63] and normal-like loudness perception [64].

### Bilateral Implantation Provides Binaural Benefits to both Adult and Child CI Users

CI devices were traditionally provided only in one ear to minimize surgical complications and cost and leave the contralateral ear amenable to future and potentially superior interventions [65]. The numerous benefits afforded to deaf children by unilateral CI stimulation are accompanied by serious limitations, especially in the domain of binaural hearing, which is essential for accurate speech detection in noise and sound localization. Unilateral hearing delays speech and language development, resulting in educational problems and feelings of embarrassment and helplessness [66,67]. Bilateral implants were first provided to adults with bilateral severe-to-profound hearing loss to promote symmetric auditory development and binaural hearing. Over the past 10 years, bilateral implantation has improved speech detection in noise, binaural sensitivity, and sound localization in adult users [10,68–71] mainly by preserving ILD cues [27,72,73]. Compared with adult users, CI children who were deaf in childhood have considerably less, if any, pre-implant acoustic experience, which means that they will have to develop perception of binaural cues following implantation.

Whether in the classroom or on the playground, children must localize and identify multiple sound sources in challenging or noisy listening environments. Benefits of bilateral implantation in children include improved speech detection in noise [11,74], speech perception in noise [8,13,75], sound localization [9,14,76], and lateralization [15]. These improvements are greater when the first CI is provided early (before the age of 2) [12] and when the second implant is provided less than 2 years after the first [16,77]. The finding that binaural difference amplitudes were largest in the absence of mismatched place of stimulation or perceived balance (ie. lack of lateralization) suggests that integration of binaural input may be occurring to some degree in children with bilateral CIs [4]. The symmetric development promoted by simultaneous implantation [2,5,6] may underlie enhanced speech detection in noise and sound localization abilities, relative to sequentially implanted peers [16]. In fact, abnormal strengthening of cortical activity driven by the unilaterally implanted ear was associated with poorer speech perception in the second implanted ear relative to the first [6].

### Binaural Fusion May be Compromised in Children who use Bilateral CIs

While bilateral implantation in children provides numerous listening advantages, deviations from normal still remain. Minimum audible angles were larger and more variable than in NH listeners (~20–40^o^) [9], speech detection benefits were asymmetric [16], and ITD detection was diminished [15]. Binaural summation was larger for the second device and spatial unmasking was significantly better when noise was moved to CI-2 in sequential users [16]. The finding that sequentially implanted children reported hearing from both devices simultaneously rather than one fused auditory image most clearly suggests abnormal perception of bilateral CI input [15]. Thus, binaural fusion must be assessed more systematically in order to determine whether children with bilateral CIs can achieve true (near-normal) binaural processing or instead shift their attention to the device with the better signal-to-noise ratio.

Binaural fusion may be compromised in children using bilateral CIs for a number of reasons such as the use of devices implanted in different locations, the use of electrical stimulation to convey auditory information, and the degree and length of auditory deprivation. Bilaterally implanted children may be using CIs that were inserted to unequal depths and/or are stimulating different populations of surviving nerve fibers. The depth of insertion affects word recognition scores [78] and mismatches in interaural place of stimulation or interaural/implant place differences (IPlDs) degrade ITD sensitivity [23,79] and brainstem integration measured by binaural difference amplitudes [4]. Kan and colleagues recently showed that ITD sensitivity deteriorates when IPlDs exceed 4 electrodes (3 mm in the cochlea). By contrast, ILD sensitivity remains relatively independent of IPlD [24] or regions of excitation in the two cochleae [80]. Even when place mismatches are eliminated, CI users have restricted access to fine-grained ITD cues with current speech processing schemes [22,70]. We thus hypothesized that binaural fusion would be best when interaural level cues are available.

Differences in the severity of hearing loss (or residual hearing) between the ears may further disrupt binaural processing, as better residual hearing has been associated with improved speech perception scores [20,81]. Abnormal reorganization throughout the auditory system may be a key factor contributing to shortcomings in binaural hearing and fusion with bilateral CIs. A sensitive period of about 1.5 years of unilateral implant use has been reported [6], beyond which inhibition from the side contralateral to CI-1 may be lost [6,82]. More than 2 years of unilateral CI use causes prolonged brainstem responses from CI-2 relative to CI-1 which persist for at least the first years of bilateral CI use [1,2,4]. The longer latencies of these responses from CI-2 may reflect decreased myelination, slower neural conduction, weaker synapses, and/or less synchronous activity relative to pathways driven by the first CI [83]. Similarly, at the level of the cortex, long durations of unilateral stimulation drive abnormal strengthening of pathways from the first implanted ear to the auditory cortex that are not reversed by 3–4 years of bilateral CI use [6]. Abnormal cortical activity evoked by long durations of unilateral CI stimulation may also result in increased effort [7].

### Electrical Hearing is More Effortful than Normal Hearing

Daniel Kahneman proposed two modes of thought: System 1, which subserves automatic and effortless cognition, such as simple addition, and System 2, which becomes activated to facilitate more deliberate and effortful decision-making [84–87]. Greater listening effort has been documented in school-aged children with hearing loss [88]. Listening challenges caused by abnormal CI stimulation also increase demands on working memory in children [17,18,89]. This is consistent with anecdotal reports from parents who comment that their children return from school each day feeling frustrated and exhausted from having to expend considerable focus and concentration when listening in class. Longer reaction times (RTs) in CI users may, in part, reflect disrupted frontal activity caused by widespread cortical reorganization due to sensory deprivation [6,58,90]. In particular, children with unilateral CIs and more than 10 years of auditory experience had abnormally large P2 peaks in their cortical waveforms [7]. This may indicate that listening was more cognitively demanding [91], required multisensory integration [92], and/or involved the reticular activating system [93,94]. Increased frontal activation reflecting greater effort has been observed in CI users when listening to speech and music [95,96]. Therefore, while children using bilateral CIs are able to achieve near-normal perception in some cases, in order to do so, they may need to recruit greater cognitive resources and rely more heavily upon System 2. Although some evidence suggests that bilateral implantation may reduce listening effort [18], compared with unilateral implantation, listening effort has not yet been measured objectively in a large number of children with bilateral CIs. We hypothesized that children using bilateral CIs expend additional effort, relative to NH peers, in an attempt to overcome device limitations and developmental abnormalities.

### Pupillometry Can be used to Quantify Effort

Pupillary constriction and dilation are mediated by autonomic regulation of the circular and radial fibers of the iris [97]. Kahneman and colleagues found that changes in pupil diameter appear to be the most sensitive and reliable objective measure of mental effort [98]. Changes in pupil diameter were more tightly associated with mental task difficulty than heart rate or skin conductance. The relationship between pupil diameter and mental effort has been well-documented for over a century [99–103]. Beatty concluded that task-evoked pupillary responses provide a reliable and sensitive indication of mental effort within tasks, across tasks, and across individuals [104]. Pupillary changes reflect net mental activity: the recruitment of greater mental resources translates into increases in pupil size. On cognitively demanding tasks, peak dilations as large as 20% of baseline responses typically occur 1–2 seconds after stimulus onset. Pupillometry has also been used to evaluate listening effort [86,105,106]. Therefore, changes in pupil diameter may be used to quantify the potentially elevated listening effort in children using bilateral CIs.

### Research Aims

The objective of the present study was to define the challenges associated with using bilateral CIs to restore binaural hearing in deaf children. We aimed to answer following research questions:

1. Can children with bilateral CIs achieve binaural fusion, ie. perceive one fused auditory image when presented with bilateral stimulation?
2. Does poorer binaural fusion translate into increased listening effort in children using bilateral CIs? We developed a protocol for measuring binaural fusion and listening effort in children who use bilateral CIs and provide an account of fusion and effort in this population.

## Materials and Methods

This study was conducted under the approval of the Hospital for Sick Children’s Research Ethics Board, which adheres to the Tri-Counsel Policy on the Ethical Conduct for Research Involving Humans. Written consent was obtained from parents or guardians on behalf of the minors enrolled in this study. This procedure was approved by the Research Ethics Board.

### Participants

Forty nine children participated in a binaural fusion task: 25 were deaf (mean age = 11.40 ± 3.49 years) and received bilateral Nucleus 24-channel CIs (Cochlear Corporation) and 24 were age-matched and had normal hearing (mean age = 12.06 ± 3.17 years; t(47) = 0.69, p = 0.50), with pure tone audiometric thresholds confirmed to be ≤ 20 dB HL at 250, 500, 1000, 2000, and 4000 Hz. All participants had normal or corrected-to-normal vision, had no known visual or developmental deficits, and were screened for visual acuity sufficient to distinguish the details necessary for performing the task without wearing eyeglasses.

All child CI users were recruited from the Cochlear Implant Program at the Hospital for Sick Children in Toronto (Table 1) and had bilateral severe-to-profound sensorineural hearing loss that occurred in childhood; hearing loss was progressive in 7 children. Eight children had a period of usable residual hearing (aided or unaided thresholds ≤ 40 dB HL at any two test frequencies 250–4000 Hz) prior to implantation. Duration of time-in-sound was calculated as the sum of the duration of CI experience and pre-implant residual hearing (time-in-sound = 8.97 ± 2.96 years; bilateral CI experience = 4.77 ± 2.56 years).

**Table 1:** CI Participant Demographic Information.

Child	Etiology	CI-1			CI-2		Inter-implant Delay (years)	Age at Test (years)	Bilateral CI Experience (years)
		Age(years)	Ear	Device	Age (years)	Device			
CI1	Unknown	5.57	R	24RE	7.11	24RE	1.54	12.14	4.91
CI3	Connexin26	2.27	L	24CA	5.58	24RE	3.31	12.16	6.50
CI4	Usher	1.12	L	24CS	4.90	24RE	3.77	11.80	6.85
CI5	Usher	0.73	R	24RE	1.62	24RE	0.90	9.28	7.59
CI6	Unknown	4.96	L	24CS	15.40	24RE	10.44	17.95	2.50
CI7	Unknown	1.88	R	24CA	4.82	24RE	2.94	10.96	6.07
CI8	Unknown	2.92	R	24RE	14.15	24RE	11.23	17.97	3.76
CI9	Unknown	5.03	L	24RE	9.83	24RE	4.81	10.40	0.45
CI10	Pendred	6.23	R	24RE	11.28	24RE	5.05	11.87	0.45
CI11	Connexin26	1.52	R	24CS	10.88	24RE	9.36	11.46	0.48
CI12	Unknown	6.41	R	24RE	10.02	24RE	3.61	14.61	0.48
CI13	Unknown	4.52	R	24CS	17.08	24RE	12.57	17.97	0.80
CI14	Usher	1.26	R	24CA	2.22	24RE	0.96	10.92	8.62
CI16	Unknown	0.87	Both	24RE	0.87	24RE	0	7.08	6.12
CI17	Unknown	4.05	Both	24RE	4.05	24RE	0	10.59	6.47
CI18	Unknown	1.28	Both	24RE	1.28	24RE	0	8.20	6.82
CI19	Pendred	3.08	Both	24RE	3.08	24RE	0	7.01	3.85
CI21	Connexin26	3.36	Both	24RE	3.36	24RE	0	9.88	6.44
CI22	Ototoxicity	12.15	Both	24RE	12.15	24RE	0	16.97	4.77
CI23	Connexin26	0.79	Both	24RE	0.79	24RE	0	5.95	5.08
CI25	Connexin26	0.95	Both	24CA	0.95	24CA	0	9.45	8.40
CI26	Unknown	0.99	Both	24RE	0.99	24RE	0	7.21	6.14
CI27	Unknown	3.16	Both	24RE	3.16	24RE	0	9.33	6.09
CI28	Ototoxicity	5.55	Both	24RE	5.55	24RE	0	9.97	4.33
CI29	Unknown	8.44	Both	24RE	8.44	24RE	0	13.87	5.31

Data is provided for each CI user (n = 25), including etiology, age at implantation, interimplant delay, age at test, and bilateral CI experience.

High resolution computed tomography scans confirmed normal cochlear anatomy in all but 3 children: child CI19 had a Mondini malformation (incomplete partition type II), child CI22 had an enlarged left vestibular aqueduct, and child CI27 presented with an enlarged vestibular aqueduct on the right side. Five children had GJB2 gene mutations causing deficiencies in Connexin 26 gap junction protein, while smaller subsets had Usher Syndrome (n = 3) and Pendred Syndrome (n = 2). Two children received ototoxic medications at a young age. The etiology of deafness was unknown in the remaining 13 children.

**Fig 1:**
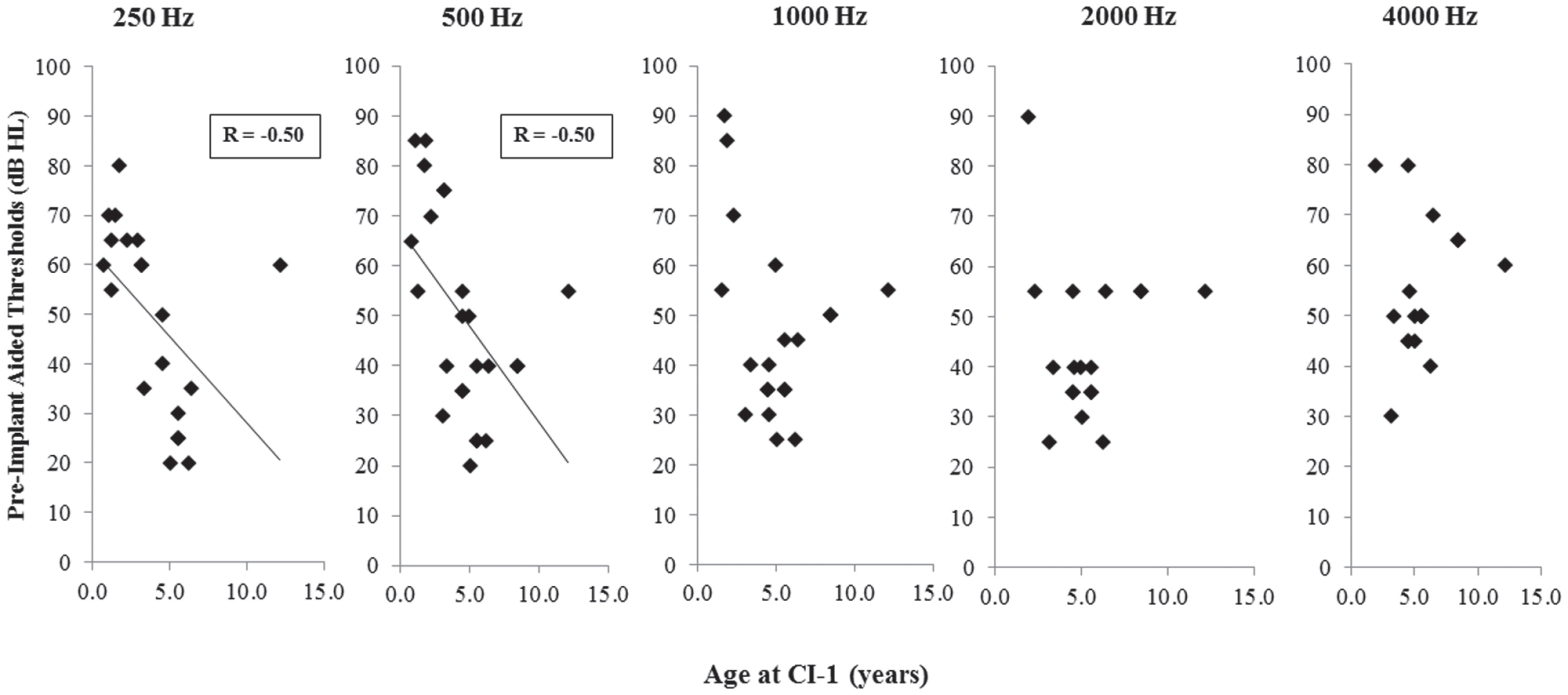
Age at CI-1 and pre-implant hearing. Children who were implanted at later ages (n = 15) had better residual hearing (ie. lower aided thresholds) at 250 Hz when assessed with standard audiometric testing prior to implantation (R = -0.50, p = 0.026). This relationship is was also present at 500 Hz (R = -0.50, p = 0.013), but not 1000 Hz (R = -0.28, p = 0.23), 2000 Hz (R = -0.01, p = 0.97), or 4000 Hz (R = 0.15, p = 0.57).

Children CI1–14 received their first devices at 3.42 ± 2.09 years of age and were provided with second devices after 5.74 ± 4.06 years of unilateral CI stimulation, whereas children CI16–29 received their implants simultaneously at 3.72 ± 3.51 years of age. Children received different device generations (Nucleus 24CA, CS, or RE) with different current conversions depending on when they were implanted. Children who had better low frequency residual bilateral hearing were implanted at older ages, as shown in Fig. 1. Significantly negative correlations were found between the age at implantation and pre-implant aided thresholds at 250 Hz (R = -0.50, p = 0.026) and 500 Hz (R = -0.50, p = 0.013). One child, CI22 was implanted at a much older age than the others (12.15 years versus 0.73–8.44 years); when data from this child was removed, these correlations were very strong: 250 Hz (R = -0.88, p < 0.0001) and 500 Hz (R = -0.65, p = 0.006). There were no significant differences between unaided thresholds prior to implantation (250 Hz: t(12) = -0.71, p = 0.49; 500 Hz: t(14) = 0.15, p = 0.89; 1000 Hz: t(8) = 0, p = 1.0; 2000 Hz: t(11) = -0.94, p = 0.37; 4000 Hz: t(7) = 0.39, p = 0.71). Correlations with age at implantation at higher frequencies were not significant: 1000 Hz (R = -0.28, p = 0.23), 2000 Hz (R = -0.01, p = 0.97), or 4000 Hz (R = 0.15, p = 0.57).

### Equipment and Stimuli

Acoustic click-trains for normal hearing listeners were presented via Matlab Version 2007b (MathWorks., Inc., Natick, Massachusetts, USA) on a Dell Vostro 1510 laptop computer and an AKAI EIE Professional soundcard (96 kHz sampling rate) through insert earphones at 250 Hz for 36 ms. One-4 trains were presented at 1 Hz. Instructions for CI stimulation were delivered via Matlab Version 2012a on a Lenovo ThinkPad Edge E420 laptop computer and a Nucleus Implant Communicator Version 2.1 system. Biphasic electrical pulse trains were presented by one of three electrodes from the apical to mid-portion of the implanted CI array (#20, 16, and 9). Each train contained pulses presented at 250 pulses per second (pps) for 36 ms and 1–4 trains were presented at 1 Hz.

Stimulus intensities were presented in CU during the fusion task to reflect the clinical environment, in which CU, instead of μA, is utilized for programming. Units were subsequently converted to dB for statistical analyses in order to compare data to NH children and to adjust for differences in current across different device generations. Units were defined by the following formulae:

dB=10log(currentinμA/100μAreference)

where μA = 10 x 175 ^CU/255^ for 24CS/CA devices and μA = 17.5 x 100 ^CU/255^ for 24RE devices.

**Fig 2:**
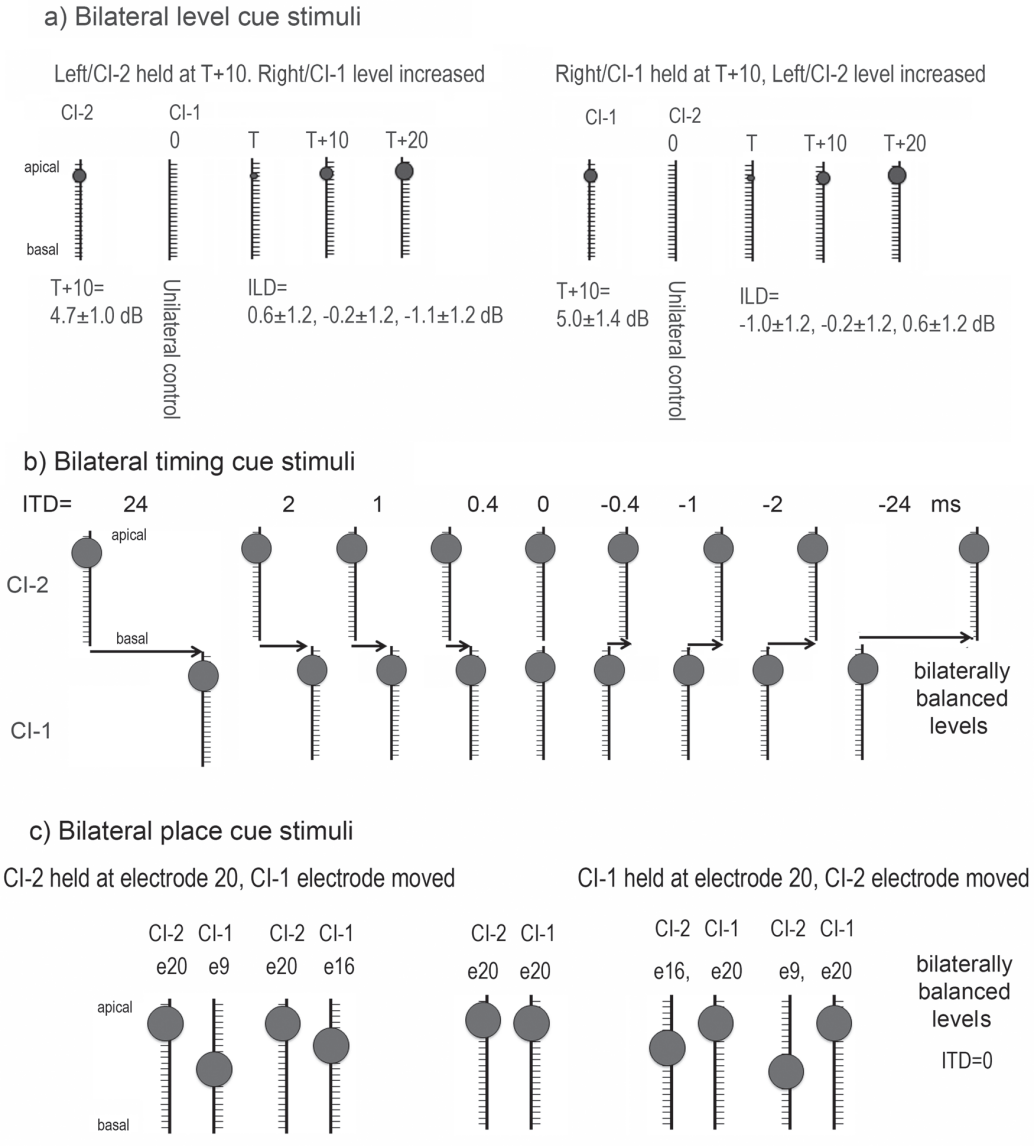
Schematic diagrams of experimental conditions. a: Bilateral level cues were presented by either holding stimulus levels constant in the left normal ear/CI-2 and increasing level in the right normal ear/CI-1 or the reverse. Increases in stimulus level are represented by the size of the circle. The circles are fairly small as levels were presented at threshold levels (T) or slightly above (T+10 or 20 dB or CU). Unilateral control conditions were also presented (T+10 in either right ear/CI-1 or left ear/CI-2 with 0 in the opposite side). Mean (±1 SD) cochlear implant unilateral stimulation levels (T+10 in each ear) and presented ILDs are shown. b) Bilateral input containing interaural/implant timing differences are shown. These were presented from e20 on both implants at levels which were comfortably loud and behaviorally balanced as shown by the larger circles at the apical end of the schematic cochlear implant array. c) Bilateral input presented at different places along the electrode array (for CI users only). Levels were comfortably loud and behaviorally balanced. The presentations were simultaneous (ITD = 0).

Stimuli in the binaural fusion task included interaural level differences (ILDs), interaural timing differences (ITDs), or interaural place differences (IPlDs). As shown in Fig. 2a, seven conditions were presented at ITD = 0 ms while ILD changed. In the panel to the left, intensity levels are held constant for CI-2 while levels are increased in CI-1 from no stimulation {T+10,0} (ie. unilateral CI-2 presentation/control stimuli) to {T+10, T}, {T+10, T+10}, {T+10, T+20}, where T = stimulation at threshold levels. On the right panel, CI-1 stimulation is held constant with increasing stimulation levels in CI-2 from no stimulation {0, T+10} (ie. unilateral CI-1 stimulation/control stimuli) to {T, T+10}, {T+10, T+10}, {T+20, T+10}, where T = stimulation at threshold level. Mean (±1 SD) ILD in dB for the CI users is shown in Fig. 2a. Note that levels were not behaviorally/centered balanced including {T+10, T+10}.

Unlike the bilateral level conditions, stimulus levels for ITD and IPlD tasks were customized for each participant with the aim of providing bilaterally centered/balanced stimulation in the middle of the dynamic range. This was accomplished by presenting levels 40 dB SPL greater than behavioral threshold for normal listeners and 33.76 ± 17.49 CU greater than behavioral threshold for CI users at electrode 20. Auditory brainstem responses had previously been recorded in all CI users at comfortably loud listening levels. Levels which evoked the most similar wave eV amplitudes on each side were reduced by 10 clinical units (CU). Pairs of electrodes were matched by location along the electrode array (ie. #20 left—#20 right; #16 left—#16 right; #9 left—#9 right). Levels were centered/balanced by asking children on which side they heard the sound. Balanced levels were defined as those which had a 50% likelihood of being perceived as coming from the right as from the left side of the head. Levels were balanced for 5 different electrode combinations {CI-2, CI-1}: {e20, e20}, {e20, e16}, {e16, e20}, {e20, e9}, and {e9, e20}. Balanced levels and behavioral thresholds are shown in Table 2 for each participant in the CI group. In Fig. 2b, 9 ITD conditions are shown: 24 ms (control stimuli), 2 ms, 1 ms, 0.4 ms, 0 ms, -0.4 ms, -1 ms, -2 ms, -24 ms (control stimuli). An additional 4 conditions were presented to the CI group in which IPlD was changed. As shown in Fig. 2c, electrode #20 was held constant either on CI-1 or CI-2 while stimulation was moved to a more basal electrode (#16 or #9).

**Table 2:** ILDs Presented to CI Users in CU and dB.

Child	Thresholds (CU)		{T+10, T}		{T, T+10}		{T+10, T+10}		{T+20, T+10}		{T+10, T+20}		ITD-VaryingTrials	
	CI-1	CI-2	CU	dB	CU	dB	CU	dB	CU	dB	CU	dB	CU	dB
CI1	190	160	-20	-1.6	-40	-3.1	-30	-2.4	-20	-1.6	-40	-3.1	-28	-2.2
CI3	185	145	-30	-1.7	-50	-3.3	-40	-2.6	-30	-1.8	-50	-3.4	-20	-1.1
CI4	145	130	-5	0.7	-25	-1.0	-15	-0.2	-5	0.6	-25	-1.1	-12	-0.4
CI5	135	135	10	0.8	-10	-0.8	0	0.0	10	0.8	-10	-0.8	-5	-0.4
CI6	165	145	-10	0.1	-30	-1.6	-20	-0.8	-10	0.0	-30	-1.7	5	1.0
CI7	170	170	10	1.6	-10	-0.1	0	0.7	10	1.5	-10	-0.2	-5	0.3
CI8	140	140	10	0.8	-10	-0.8	0	0.0	10	0.8	-10	-0.8	0	0.0
CI9	120	150	40	3.1	20	1.6	30	2.4	40	3.1	20	1.6	2	0.2
CI10	150	150	10	0.8	-10	-0.8	0	0.0	10	0.8	-10	-0.8	0	0.0
CI11	165	155	0	0.9	-20	-0.8	-10	0.0	0	0.8	-20	-0.9	-40	-2.7
CI12	120	130	20	1.6	0	0.0	10	0.8	20	1.6	0	0.0	5	0.4
CI13	130	140	20	2.8	0	1.1	10	1.9	20	2.7	0	1.0	-20	-0.9
CI14	175	160	-5	0.4	-25	-1.3	-15	-0.5	-5	0.3	-25	-1.4	-15	-0.4
CI16	155	150	5	0.4	-15	-1.2	-5	-0.4	5	0.4	-15	-1.2	0	0.0
CI17	175	145	-20	-1.6	-40	-3.1	-30	-2.4	-20	-1.6	-40	-3.1	-12	-0.9
CI18	140	150	20	1.6	0	0.0	10	0.8	20	1.6	0	0.0	-10	-0.8
CI19	155	170	25	2.0	5	0.4	15	1.2	25	2.0	5	0.4	-18	-1.4
CI21	160	150	0	0.0	-20	-1.6	-10	-0.8	0	0.0	-20	-1.6	-13	-1.0
CI22	140	135	5	0.4	-15	-1.2	-5	-0.4	5	0.4	-15	-1.2	3	0.2
CI23	140	140	10	0.8	-10	-0.8	0	0.0	10	0.8	-10	-0.8	20	1.6
CI25	175	175	10	0.9	-10	-0.9	0	0.0	10	0.9	-10	-0.9	3	0.3
CI26	160	155	5	0.4	-15	-1.2	-5	-0.4	5	0.4	-15	-1.2	-10	-0.8
CI27	150	150	10	0.8	-10	-0.8	0	0.0	10	0.8	-10	-0.8	0	0.0
CI28	135	125	0	0.0	-20	-1.6	-10	-0.8	0	0.0	-20	-1.6	10	0.8
CI29	150	125	-15	-1.2	-35	-2.7	-25	-2.0	-15	-1.2	-35	-2.7	-2	-0.2

For all CI children who participated in the fusion task, thresholds are shown in CU, as well as the ILDs presented in various conditions in terms of both CU and dB.

### Binaural Fusion Task

Children were asked to indicate whether they heard 1 solid sound or 2 separate sounds in a two-alternative forced choice test by clicking on a single circle or pair of circles on a laptop monitor as fast as possible. In a training session, unilaterally presented stimuli (1 solid sound) and binaural stimuli presented at large ITDs (±24 ms) (2 separate sounds) were presented with feedback. These control stimuli were incorporated into the test stimuli in random order. Stimuli were presented in a randomized block design. Children with normal hearing completed 10 blocks of 16 conditions (160 total trials) and children with bilateral CIs completed 8 blocks of 20 conditions (172 total trials). Children who responded with < 70% accuracy [15] to control stimuli were excluded from data analyses.

### Reaction Times and Pupillometry

Reaction times (RTs) were recorded simultaneously from stimulus onset to response. Outliers in RT (either > 3 SDs of the mean or < 250 ms) [103] were excluded from analyses. Mean RTs longer than 7 s were no longer considered valid as responses to the stimulus. Pupil data from each participant’s better eye (ie. the eye with the smaller number of missing data points) were measured [103] at 105 Hz using an Interacoustics VN415/VO425 Videonystagmography (VNG) system (DK-5610, Assens, Denmark) and relative pupil size was indicated on the CCD camera chip. The percent of change in pupillary diameter (PCPD) was calculated relative to baseline values, as reported previously with similar measurement equipment [107]. Outliers in PCPD (greater/less than 3 SDs of the mean) were also excluded. For each stimulus trial, the FeatureFinder Version 2.5 program was used in Matlab to determine the peak pupil diameter automatically during the first 2 seconds following stimulus onset in order to control for large differences in RTs across participants. Baseline pupil data recorded during the 1 s preceding stimulus presentation, while participants fixated on a plain screen, was subtracted from the peak diameter for each trial to control for individual differences in pupil size. Monitor brightness and room lighting were held constant through the task. Participants were instructed to inhibit blinks in between trials. Uninhibited blinks were linearly interpolated and few trials (approximately 20%) were removed following trial-by-trial visual inspection of the pupil waveform for excessive blinking or error [106]. Pupil data from 9 participants (5 NH, 4 CI) were excluded due to significant error resulting from excessive eye movement and/or poor camera focus.

### Electrically Evoked Auditory Brainstem Responses

EABRs recorded 2.16 ± 1.24 years previously in each child were compared to behavioral fusion results from the present study. EABRs were evoked in 24 CI users by biphasic pulses delivered from electrode 20 at 11 Hz using a SPEAR processor (in collaboration with CRC-HEAR, Melbourne, Australia) and were measured at a midline cephalic location (Cz) referenced to the ipsilateral earlobe. Data were collected using a Neuroscan system (NSI, Virginia, USA, V4.3) and Synamp I (AC/DC) amplifier. At least 300 sweeps were filtered (10–3000 Hz) in a-5–80 ms time window and averaged for each stimulus presentation level; those sweeps with amplitudes ±30 μV were rejected from the average. A minimum of two visually replicable averages were obtained at each presented intensity. This procedure has previously been described in more detail [108]. Latencies of the largest and most persistent peak (wave eV) were measured by two independent markers with very good inter-rater reliability (ICC = 0.86).

### Data Analysis

The proportions of “1” responses to ILDs, ITDs, and IPlDs were analysed for each child using binary logistic regression. Separate analyses were conducted by ear of ILD or IPlD manipulation. Similarly, responses to ITDs leading from the left ear were analysed separately from responses to ITDs leading from the right. Repeated-measures Analysis of Variance (ANOVA), Chi-Square (χ^2^) Tests, Student’s T Tests, and regression were conducted using IBM SPSS Statistics Version 22 (SPSS Inc., Chicago, Illinois, USA). Linear regression analyses were performed for all measures to determine whether demographic factors of interest (age at CI-1, interimplant delay, bilateral CI experience, time-in-sound) carried any predictive value. Pairwise post-hoc analyses were implemented for repeated contrasts and Bonferroni adjustment of the significance level (α = p = 0.05) was used where necessary to correct for multiple comparisons and limit the family-wise error rate.

## Results

**Fig 3:**
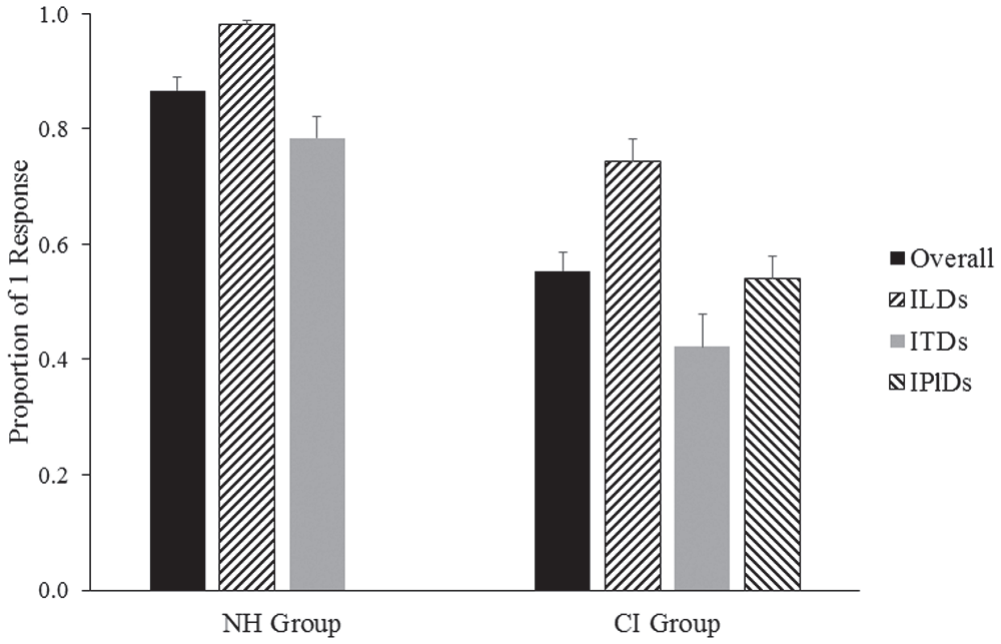
Mean (± 1SE) proportions of 1 response across all conditions are shown by the black bars. Overall CI listeners (n = 25 perceived 1 sound less frequency than NH peers (n = 24; p<0.0001). Mean (± 1SE) proportions of 1 response are also shown across each of the subtest conditions (ILDs = interaural/implant level differences; ITDs = interaural/implant timing differences; IPlDs = interaural/implant place of stimulation differences) tested in each group. In both groups, ILDs were most frequent perceived as fused and ITDs were least often perceived as fused (p<0.0001).

Overall mean proportions of responses from all children for the binaural fusion task are shown in Fig. 3. Normal listeners perceived one fused auditory image more frequently than their peers with bilateral CIs (χ^2^(1) = 606.20, p < 0.0001). In both groups, ITDs were significantly poorer cues to fusion than ILDs (NH group: χ^2^(1) = 282.42, p < 0.0001; CI group: χ^2^(2) = 192.31, p < 0.0001) and were associated with the most variability in responses (NH SD = 0.18; CI SD = 0.29). Mean performance for trials varying in terms of IPlDs fell in between mean performance for trials containing an ILD or ITD.

### Fusion with Level Cues

**Fig 4:**
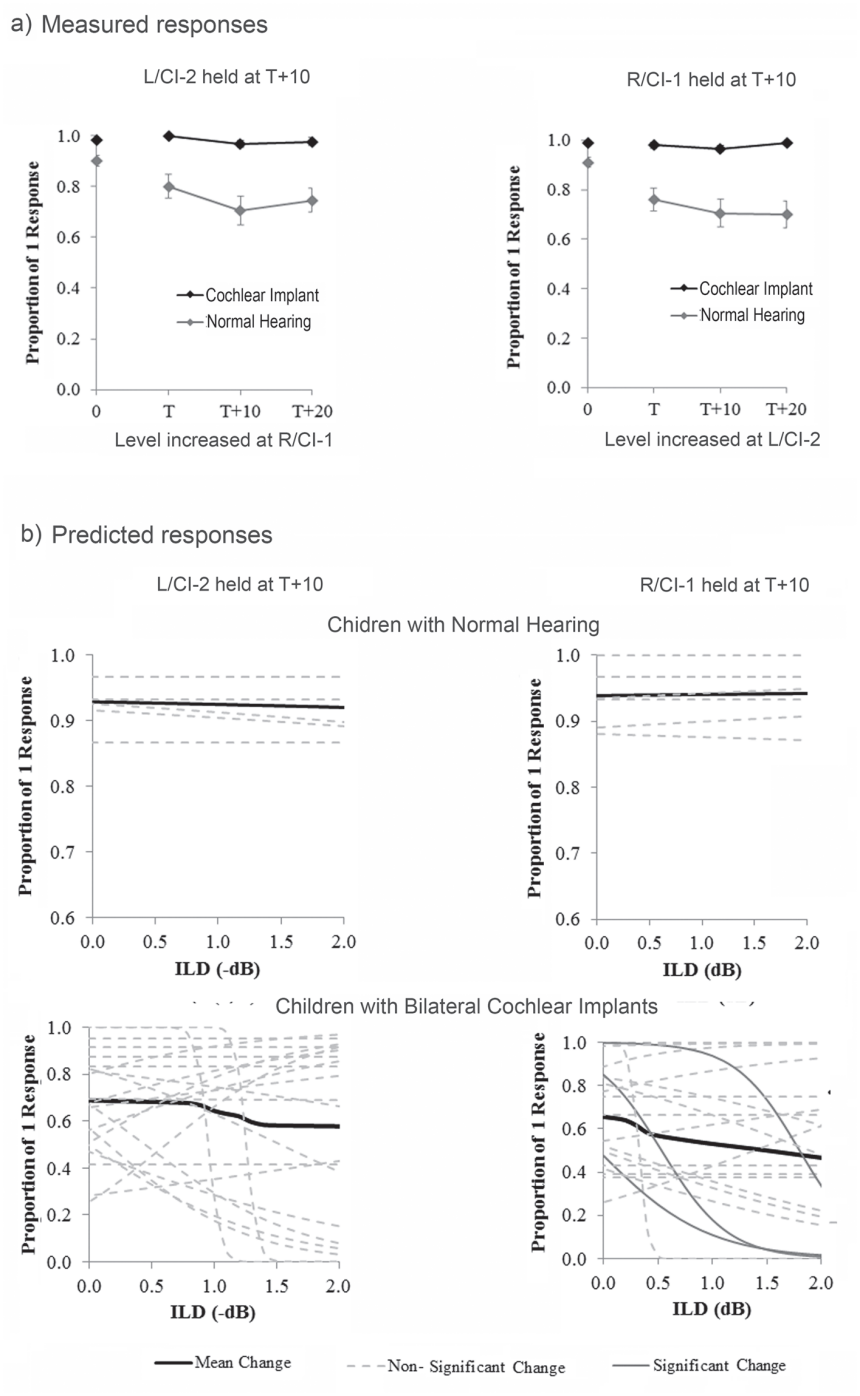
Fusion with interaural level differences. a) Group performance for conditions containing ILDs (ITD = 0 ms). Biphasic pulses were delivered from electrode 20 in the CI group (n = 25). CI listeners consistently perceived one image when there were level differences, albeit less frequently than NH peers (n = 24; p < 0.0001). b) Binaural fusion was predicted as a function of ILD for individual normal hearing children and CI users with logistic regression. None of the slopes were significant in the normal hearing children as shown by the dashed lines (p > 0.05). For CI users, the majority of curves tend to decrease as a function of increasing ILD. Significant slopes (n = 3) are represented by dark grey solid lines.

Fig. 4a shows mean responses in both groups to ILD-varying conditions (ITD = 0 ms). Responses to unilateral control conditions {T+10,0}, {0, T+10}, where T = threshold, are displayed on the y-axis (NH right (R) level changing = 0.98 ± 0.05; NH left (L) level changing = 0.99 ± 0.04; C1–1 level changing = 0.89 ± 0.12; CI-2 level changing = 0.91 ± 0.10). Perception of 1 sound on at least 70% of unilateral trials [15] was taken as an indication of task comprehension and all children included in data analyses met this criterion. For the left panel in Fig. 4a, fusion of binaural input with level cues is represented as a function of changes in the level provided from CI-1 (or the right side), relative to CI-1 threshold (T), with the level provided from CI-2 (or the left side) held constant at 10 CU or dB above CI-2 or L threshold. Similarly, for the right panel in Fig. 4a, fusion is shown as a function of changes in level provided from CI-2, with the level delivered from CI-1 held constant at 10 CU above the CI-1 threshold. Normal listeners consistently perceived one fused auditory image (98 ± 4% of trials) when no ITD was introduced. While CI users perceived one image less frequently than NH listeners when ILDs were present (74 ± 19% of trials; χ^2^(1) = 869.81, p < 0.0001), they still met control criteria (≥ 70%) on each condition and therefore appeared to be perceiving fused auditory images, at least, as a group.

Logistic regression was used to predict changes across conditions for each participant in both groups for the dichotomous outcome variable (Fig. 4b). Responses are shown for both sides separately as a function of ILD. All regression functions had relatively horizontal slopes for the NH group (p > 0.05) with values well above 0.7 (control criterion) when level was increased on either side. To allow for between-group comparisons, the independent variable (ILD) was transformed to dB for the CI group (dB = 10 log (current in μA / 100 μA). Significant functions are shown in dark grey and were found for only three children (CI11, CI16, and CI19) when CI-2 level was changed. Of the 3 CI users with significant responses to changes in ILD: two (CI11 and CI19) had shorter durations of bilateral CI experience (0.48 and 3.85 years, respectively), while the third child (CI16) had inconsistent bilateral CI use over the first few years of activation.

### Fusion with Timing Cues

**Fig 5:**
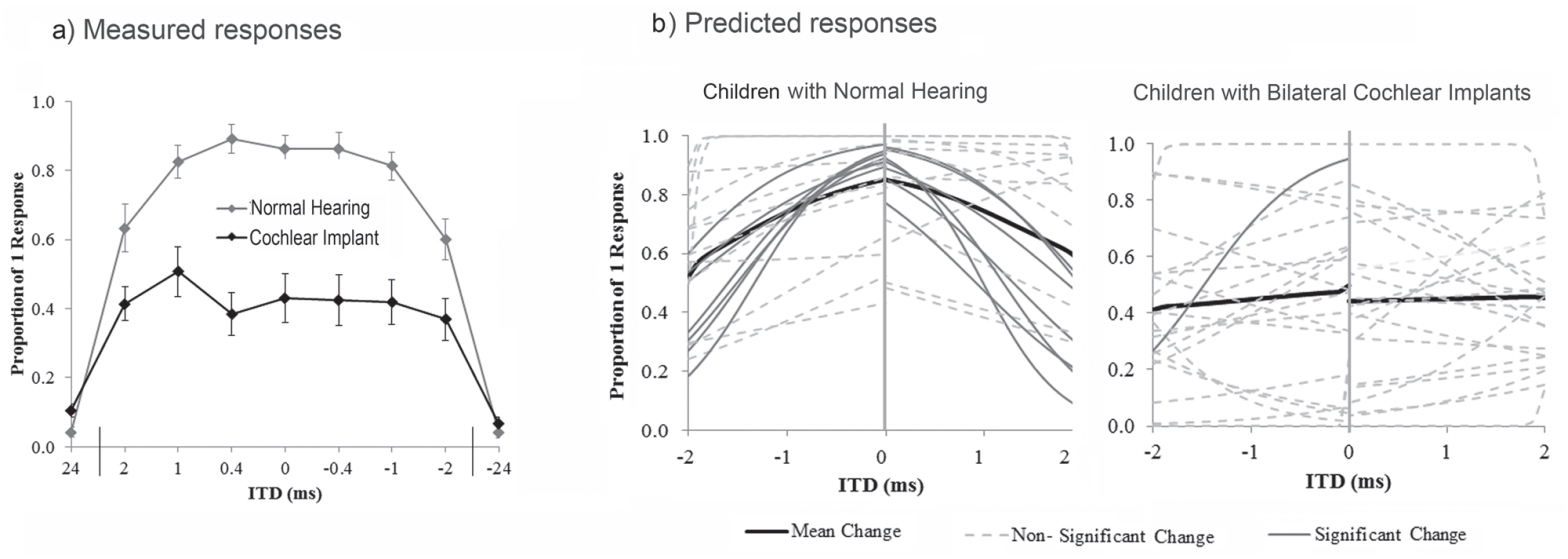
Fusion with interaural timing differences. a) Mean responses in children with normal hearing (n = 24) and cochlear implants (n = 25) for conditions with varying ITDs (interaural/implant timing differences). Balanced stimuli presented for ITD-varying trials in the CI group contained a small mean ILD of-0.34 ± 0.90 dB. Children with normal hearing were more likely to hear two separate sounds as the ITD increased to ±2 ms (p < 0.0001), while CI users were not (p > 0.05). Negative values denote R/CI-1 leading ITDs. b) Individual regression functions are plotted across ITDs ranging from-2 to 2 ms. As shown in the bottom plot, ITDs did not affect fusion in 24/25 CI users (p > 0.05).

Fig. 5a shows mean responses in both groups for ITD-varying conditions. CI users received CI stimulation from electrode 20. Responses to the ±24 ms control conditions are indicated on the extreme ends of the x-axis (NH R-leading = 0.04 ± 0.08; NH L-leading = 0.04 ± 0.07; C1–1 leading = 0.07 ± 0.09; CI-2 leading = 0.10 ± 0.09). When presented with balanced levels, NH listeners perceived one image on 78 ± 18% of trials. Conversely, CI users reported hearing one sound almost half as frequently as their NH peers (42 ± 29% of trials) when level cues were removed (χ^2^(1) = 308.03, p < 0.0001). Furthermore, NH children were more likely to hear two separate sounds as the ITD was increased beyond ±1 ms (L-lead: χ^2^(1) = 20.75, p < 0.0001; R-lead: χ^2^(1) = 21.01, p < 0.0001), in contrast to CI children who did not demonstrate any significant changes in response to increases in ITD beyond this range (CI-2 leading: χ^2^(1) = 1.94, p = 0.16; CI-1 leading: χ^2^(1) = 0.73, p = 0.39).

Logistic regression functions are shown in Fig. 5b for individual participants: 10/24 normal listeners exhibited significant changes in perception with increasing ITDs on at least one side. There was no age difference between NH listeners with significant and non-significant responses (L-leading ITDs: t(22) = -0.53, p = 0.60; R-leading ITDs: t(22) = -0.43, p = 0.67). In the absence of level or place cues, ITDs did not systematically affect binaural fusion in the CI group, as only one child using CIs exhibited significant changes in perception of 1 sound with changes in ITDs on either side.

### Fusion with Place of Stimulation Cues

**Fig 6:**
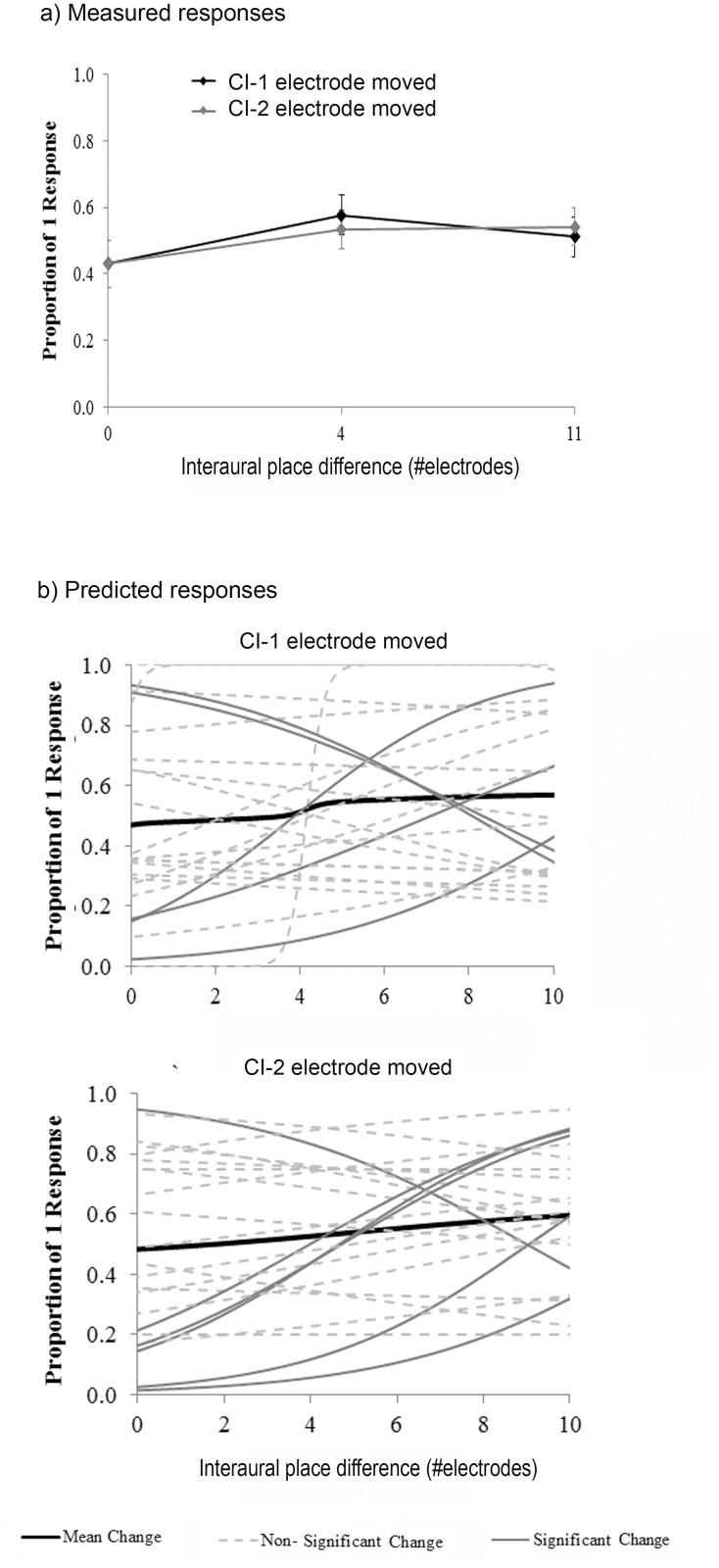
Fusion with interaural place of stimulation differences. a) Mean responses in the CI group (n = 25) are displayed as a function of increasing difference in the place of stimulation (IPlD) between sides. Place of stimulation was held constant at electrode 20 on one side while pulses were delivered from more basal electrodes on the contralateral side (electrode 16 for IPlD = 4 and electrode 9 for IPlD = 11). b) IPlDs did not affect fusion in 16/25 children with CIs (p > 0.05).

As a group, CI listeners performed at chance (mean proportion = 0.54 ± 0.19) when no level or timing differences were present and only place of stimulation was changed (Fig. 6a). As plotted in Fig. 6b, 9/25 CI users showed significant changes in perception when the electrode position was varied on at least one side, but as in the case of ITDs, these changes were not consistent or systematic, because only 2 children had significant changes in response to variations in both CI-1 and CI-2 with changing interaural place differences (IPlDs) of stimulation.

### Predicting Binaural Fusion

**Fig 7:**
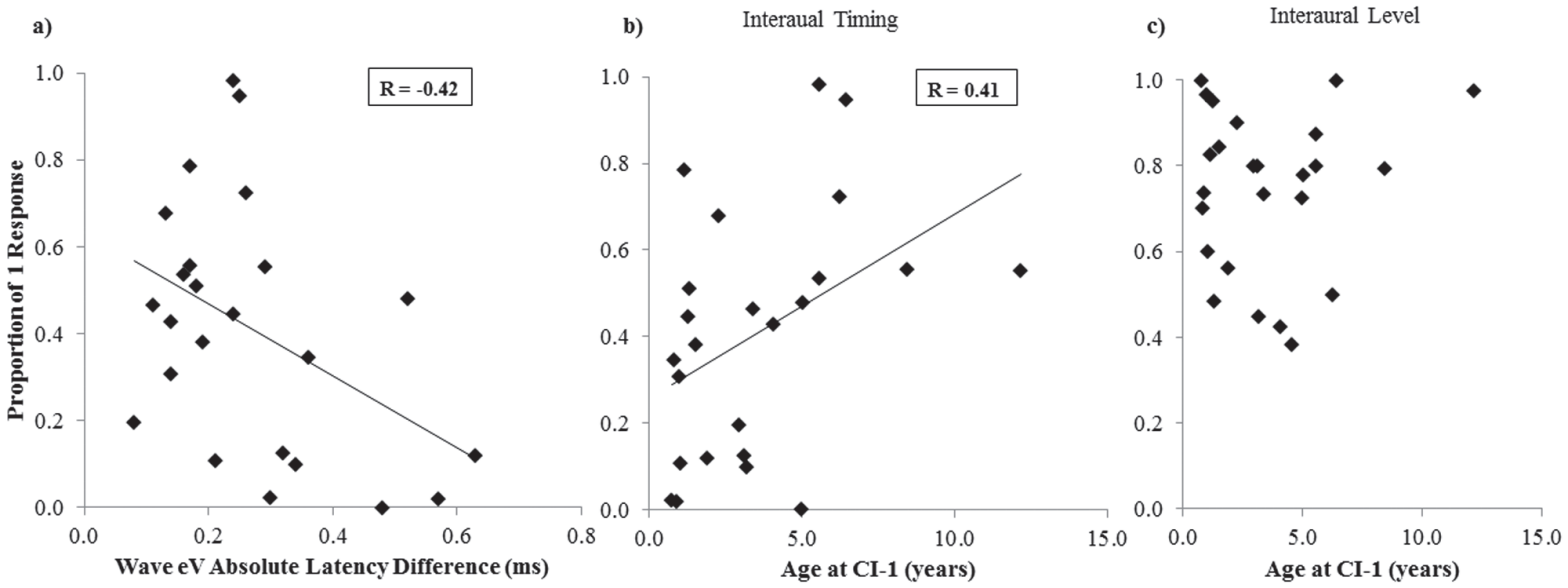
Predicting binaural fusion. Multiple regression analysis revealed that mean proportion of 1 response for individual CI users (n = 24) when level and place differences were absent can be predicted (p < 0.05) by a) absolute wave eV latency difference (β = -0.42) and b) the age at CI-1 (β = 0.41). c): There was no relationship between age at CI-1 and fusion with level differences (p > 0.05).

Stepwise multiple linear regression analysis was used to assess predictors of binaural fusion. Age at CI-1, age at test, interimplant delay, bilateral CI experience, duration of deafness, time-in-sound, and absolute EABR wave eV mismatch were included as independent variables with binaural fusion in the absence of level or place of stimulation cues as the dependent outcome variable. The analysis revealed that age at CI-1 and absolute EABR wave eV mismatch best predict further lack of fusion in the absence of level or place of stimulation cues (R = 0.60, p = 0.01; Fig. 7a: β for wave eV mismatch = -0.42, p = 0.03; Fig. 7b: β for age at CI-1 = 0.41, p = 0.03). Children with exceptionally large EABR mismatches (> 0.5 ms) had longer interimplant delays and/or less/inconsistent bilateral CI use.

### Reaction Time

**Fig 8:**
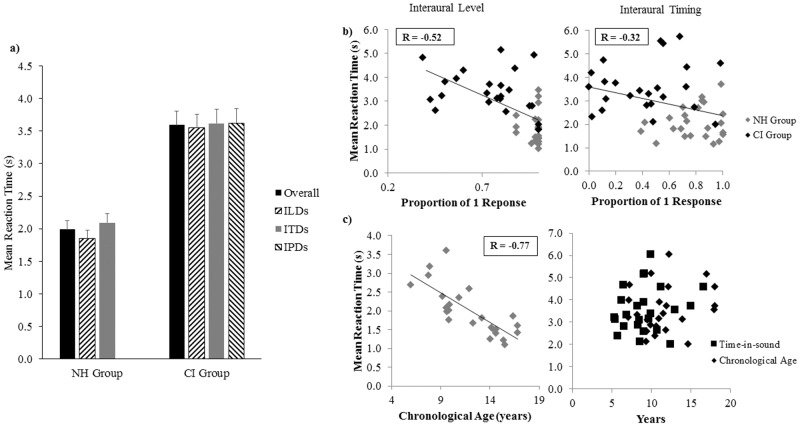
Fusion and reaction time. a) Mean overall RTs (reaction times) and RTs for each subset of conditions (ILDs = interaural/implant level differences; ITDs = interaural/implant timing differences; IPlDs = interaural/implant place of stimulation differences) are displayed for both groups. CI users (n = 24) had longer RTs than their peers with NH (n = 24; p < 0.0001). b) Mean RTs are shown for individual participants. Poorer fusion predicts longer RTs (level cues: R = -0.52, p < 0.001; timing cues: R = -0.32, p < 0.05). c) In the NH group, children achieved faster RTs at older ages (R = -0.77, p < 0.0001), while there was no relationship between RT and chronological age or duration of time-in-sound in the CI group (p > 0.05).

CI users had longer overall reaction times (RTs) than their NH peers (Fig. 8a; t(38.80) = -6.45, p < 0.0001). Factorial repeated-measures ANOVA revealed main effects of group on RTs for conditions with R/CI-1 level changing (F(1,46) = 40.51, p < 0.0001), L/CI-2 level changing (F(1,46) = 45.19, p < 0.0001), and ITD changing (F(1,46) = 35.47, p < 0.0001). In the NH group, RTs were longer with respect to some conditions with larger ITDs (F(6,138) = 2.66, p = 0.02; 0.4 ms ITD vs. -2 ms ITD: p = 0.02) and smaller ILDs (R changing: F(2,46) = 1.92, p = 0.16; L changing: F(2,46) = 3.55, p = 0.04; {T, T+10} vs. {T+20, T+10}: p = 0.04), but these differences were not statistically significant after correcting for multiple comparisons. Similarly for the CI group, there was no effect of any subset of conditions on RT (ITD: F(6,138) = 0.72, p = 0.63; CI-1 level changing: F(2,46) = 0.51, p = 0.61; CI-2 level changing: F(2,46) = 1.11, p = 0.34; CI-1 place changing: F(2,46) = 0.44, p = 0.65; CI-2 place changing: F(2,46) = 0.90, p = 0.41).

As shown in Fig. 8b, longer mean RTs were significantly associated with poorer fusion (interaural level cues: R = -0.52, p < 0.001; interaural timing cues: R = -0.32, p = 0.03) and younger ages in the NH group (Fig. 8c: R = -0.77, p < 0.0001). No demographic factor predicted RTs of CI children (age at CI-1: R = 0.18, p = 0.40; interimplant delay: R = 0.08, p = 0.73; bilateral CI experience: R = 0.01, p = 0.97; time-in-sound: R = 0.18, p = 0.40; chronological age: R = 0.19, p = 0.34).

### Pupillary Responses

**Fig 9:**
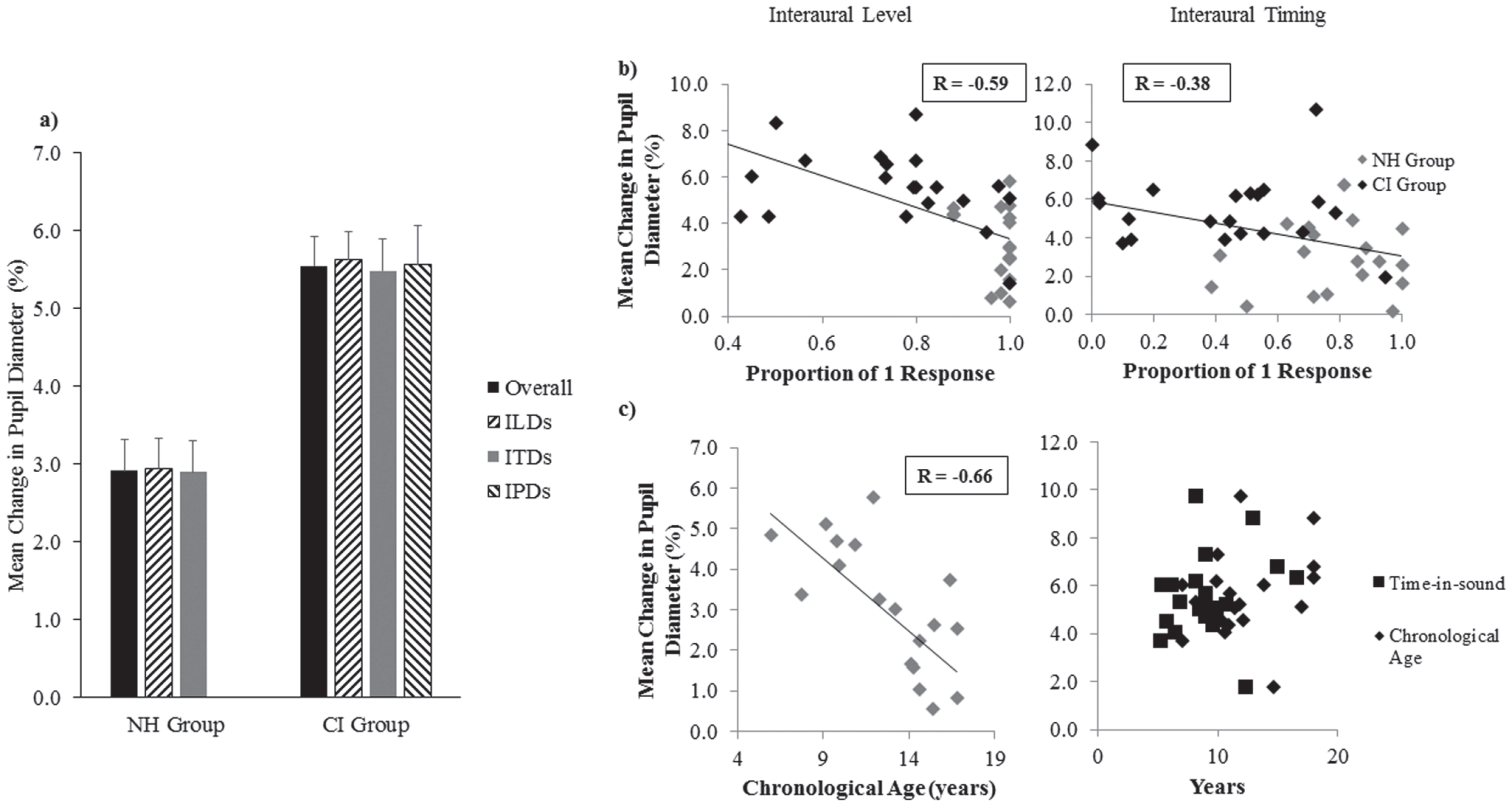
Fusion and pupil diameter. a) Mean PCPD (percent of change in pupillary diameter) is shown for each group. Children with bilateral CIs (n = 21) had greater changes in pupillary diameter than their NH peers (n = 19; p < 0.0001). b) Greater changes in pupil diameter were associated with poorer binaural fusion (level cues: R = -0.59, p < 0.0001; timing cues: R = -0.38, p < 0.05). c) Older NH children had smaller changes in pupil diameter, reflecting less listening effort (R = -0.66, p < 0.005), while there was no relationship between pupil diameter and time-in-sound or chronological age in the CI group (p > 0.05).

While latencies peak pupil dilation were similar between groups (t(38) = -0.98, p = 0.34) and consistent with pupil physiology [106], CI users had greater overall changes (ie. differences between baseline and peak dilation) than their NH peers (Fig. 9a; t(38) = -4.84, p < 0.0001) and when each interaural cue was varied (R/CI-1 level changing: F(1,38) = 17.54, p < 0.0005; L/CI-2 level changing: F(1,38) = 29.94, p < 0.0001; timing: F(1,38) = 20.60, p < 0.0001). In both groups, PCPD (percent of change in pupillary diameter) did not differ across ITDs (NH: F(6,108) = 1.62, p = 0.15; CI: F(6,120) = 0.62, p = 0.72) or ILDs (R changing: F(2,36) = 0.01, p = 0.99; CI-1 changing: F(2,40) = 3.32, p = 0.05 (ns); L changing: F(2,36) = 0.32, p = 0.73; CI-2 changing: F(2,40) = 2.79, p = 0.07). Additionally, pupillary responses did not change significantly across IPlDs (CI-1 changing: F(2,40) = 2.00, p = 0.15; CI-2 changing: F(2,40) = 0.45, p = 0.64) or subsets of interaural cue conditions (NH: t(18) = -0.16, p = 0.87; CI: F(2,40) = 0.12, p = 0.89).

**Fig 10:**
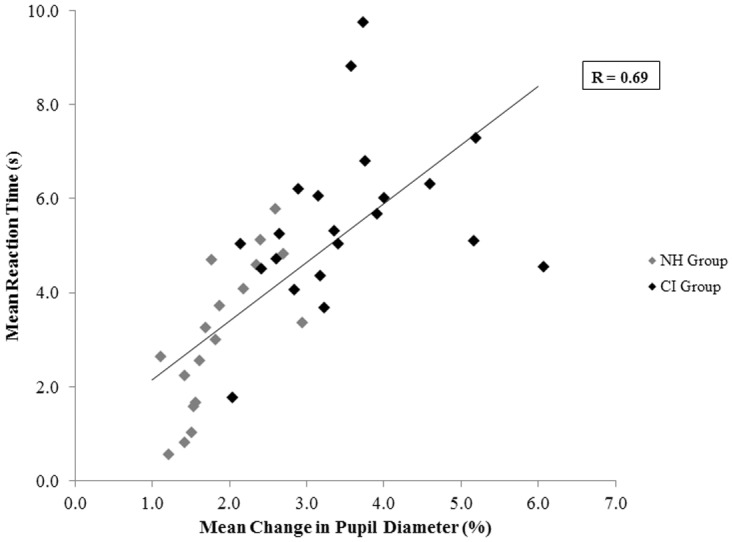
Reaction time and pupil diameter. Mean overall pupillary responses were positively correlated with RTs (R = 0.69, p < 0.0001; n = 40).

For all children across all conditions, mean PCPD showed a similar trend to RTs and increased with poorer fusion (Fig. 9b—level cues: R = -0.59, p < 0.0001; timing cues: R = -0.38, p = 0.01). Older NH children displayed smaller PCPDs (Fig. 9c; R = -0.66, p < 0.005), whereas no demographic factor was related to pupillary responses in the CI group (age at CI-1: R = 0.13, p = 0.59; interimplant delay: R = 0.35, p = 0.12; bilateral CI experience: R = 0.18, p = 0.44; time-in-sound: R = 0.18, p = 0.44; chronological age: R = 0.27, p = 0.24). Not surprisingly, as illustrated in Fig. 10, greater PCPDs predicted longer RTs (R = 0.69, p < 0.0001).

## Discussion

The main objective of this study was to determine whether children who are deaf are able to perceptually integrate or fuse sounds from two different CI devices. We were also interested in whether listening effort in CI users would increase as binaural fusion decreased.

Across all conditions, CI users perceived one auditory image (ie. “fused” bilateral input) less frequently than their NH peers. Bilateral input was more often perceived as fused when interaural level difference (ILDs) were present than when levels were balanced and interaural timing differences (ITDs) were present. Larger asymmetries at the level of the auditory brainstem translated into even poorer binaural fusion in the CI group. Better binaural fusion was associated with longer acoustic experience before cochlear implantation which might have primed the system for processing bilateral CI stimulation. A reduced ability to fuse bilateral input resulted in increased reaction times (RTs) and pupil diameter, reflecting increased listening effort in CI users. RTs and pupillary changes decreased with age in NH children.

Compared to NH peers, binaural fusion was found to be abnormal in children using bilateral CIs (Fig. 3). When both ears are stimulated with acoustic input within an ITD of ±1 ms, normal listeners were able to perceive one fused auditory image (Fig. 5a). By contrast, children using bilateral CIs listen to bilateral input through two different devices placed in different cochlear locations possibly stimulating different complements of surviving auditory neurons with pulsatile electrical stimulation. All of these issues could explain the findings of impaired binaural fusion in child bilateral CI users [22,23,90]. Furthermore, periods of unilateral and bilateral deprivation cause abnormal reorganization throughout the auditory pathway [2,3,6,7,51,58] which may further limit the ability of the central nervous system to fuse binaural input.

### Fusion is Impaired in Children with Bilateral CIs

Results from the present study suggest that children with bilateral CIs do not have the same binaural processing as their normal hearing peers. Perception of bilateral input as one sound increased with access to interaural level cues and longer acoustic hearing prior to implantation. The present cohort of cochlear implant users generally perceived two distinct auditory images (overall mean data shown in Fig. 3). This is consistent with findings of Salloum and colleagues who reported that sequentially implanted CI users rarely heard a single sound in the middle of their heads in the absence of ILDs or ITDs, in contrast to a control group of normal hearing peers [15]. Thus, children with bilateral CIs may not localize unitary auditory images, but rather have learned to use binaural cues to attend to the more salient of two monaural images.

Results from adult bilateral implant users suggest that impaired binaural fusion is particular to children with early onset deafness. Adult CI users heard a single fused auditory image from their bilateral implants on a larger proportion of trials than the cohort of children studied here [24]. The difference is likely attributable to the considerably greater pre-implant bilateral acoustic experience in the adults relative to the pediatric cohorts. Longer periods of acoustic hearing prior to implantation may promote fusion with bilateral CIs by developing the neural pathways in the auditory system that mediate binaural fusion. In support, binaural fusion of input containing interaural timing or place of stimulation cues improved as age at implantation increased (Fig. 7b). It is clear that earlier ages at implantation are recommended in the case of profound congenital deafness to promote normal development of speech perception skills [109] and maintain the integrity of the auditory system. With that in mind, implantation at later ages presently occurs in children who had better hearing. As shown in Fig. 1, increasing age at implantation was associated with lower pre-implant pure tone audiometric thresholds with hearing aids, indicating better access to bilateral acoustic sounds. This pre-implant experience did not benefit post-implant ILD perception (Fig. 7c). A similar finding was shown in adult CI users by Litovsky and colleagues [71]. In contrast to ILDs, perception of ITDs is based on information conveyed in the fine structure of acoustic waveforms [110]. Fine structure information is less relevant for coding ILDs, which are carried mainly in the temporal envelope [80] and are represented more faithfully by current envelope-based CI stimulation strategies. Consistent with the positive effect of acoustic experience shown in the present study, pre-implant hearing has also been found to enhance post-implant sound localization and music perception in CI children [12,111] and speech perception, music perception, and ITD sensitivity in post-lingually deafened adults with CIs [71,112,113]. Greater residual hearing also appears to provide important developmental benefits [55]. Thus, ILD processing may be more robust to hearing loss, may not require exposure to bilateral acoustic input, or may have different mechanisms than those required for coding ITDs.

As confirmed by the present study, large ILDs do not interfere with binaural fusion in normal hearing listeners [25], but rather shift perception of a fused image to the contralateral side (Fig. 4b), presumably with increasing contralateral inhibition in the LSO [114]. Conversely, in the absence of ILDs, ITDs extended beyond the physiological range (ie. the ±2 ms stimuli) increase the likelihood of perceiving two separate auditory images (Fig. 5a; L-leading: χ^2^(1) = 20.75, p < 0.0001; R-leading: χ^2^(1) = 21.01, p < 0.0001). Although children with bilateral CIs were generally less likely to perceive a fused image, they were also more successful at this task when level cues were available (Fig. 4a; χ^2^(2) = 192.31, p < 0.0001). Recent observations that ITD perception is more easily disrupted by interaural frequency or place mismatches than ILD perception in both NH individuals and those with bilateral CIs support the notion that binaural input with level cues is more effectively fused by the auditory system [24,37]. Thus, ILD processing may not require similar regions to be excited within each ear and instead occurs via a different neural mechanism than ITD processing [80]. As shown by previous studies, CI users were less sensitive to bilateral input containing large IPlDs [4,69] and were biased towards the side with more basal stimulation [24].

Perception as a function of ITD differs when children are asked to fuse rather than lateralize binaural input. Many of the children with CIs studied here were able to lateralize sounds on the basis of ITDs in a separate study [115]; however, only one fused binaural input similarly to the normal hearing group. Similarly, normal listeners can detect changes in ITDs as small as 10 μs, but require very large ITDs, as much as ±2 ms, to lose binaural fusion [116]. The data thus indicate that multiple psychophysical ranges may exist: one for lateralization and a different one for fusion. Kan and colleagues also found that fusion and lateralization were not directly related [24]. Different neural codes may underlie fusion and lateralization [35,36,41]. Although lateralization is normally processed through the superior olivary complex of the brainstem, evidence from patients with pontine lesions suggests that a more direct lemniscal pathway to the inferior colliculus can take over albeit with impaired sensitivity [117].

### Poorer Binaural Fusion Increases Task Difficulty

Children in the CI group showed increased reaction times (RTs) than NH peers during the binaural fusion task (t(38.80) = -6.45, p < 0.0001) and greater mean differences between baseline and peak pupil dilation (t(38) = -4.84, p < 0.0001). The observed difference in these measures between the groups may be mediated largely by the fact that impaired hearing, in general, is more effortful [88]. However, the associations between RT and percent change of pupillary diameter (PCPD) with the proportion of 1 response across all children tested (Figs. 8b and 9b, respectively) suggest that a poorer ability to perceive a fused image is indicative of a general increase in task difficulty which could also require increased listening effort. The fusion of two degraded percepts may elicit additional mental computation and uncertainty that makes bottom-up processing even more effortful for CI users. In contrast to children who consistently perceive one fused image and can more automatically and effortlessly localize or interpret the simpler unitary percept using System 1 defined by Kahneman [84], those who more frequently perceive distinct images must first dedicate more time and mental resources (System 2) [84] to deciding whether they hear one or two sounds. These children must then determine which features of each image should receive attention and be sent for further processing in higher centers. Inhibition of top-down repair mechanisms may further complicate bottom-up processing in CI users [87].

If the perception of two separate images itself was the main reason for observed increases in effort, then conditions in which children consistently perceived two distinct images should have been associated with significantly greater increases in effort. However, mean pupillary responses were not the largest for the ±24 ms ITD control conditions in which all children consistently (~90% of trials) perceived two separate sounds. In support of this line of reasoning, Kahneman and Beatty observed that pupillary changes were the smallest when it was clearest to participants that two different tones were presented in a pitch discrimination task [86]. Pupil diameters increased in size with increasing difficulty of the discrimination. The findings from the present study thus suggest that poorer fusion results in greater listening effort because of the mentally taxing decision-making process involved in determining whether one or two sounds were heard prior to response selection. The correlation between RTs and PCPDs supports this assertion (Fig. 10): greater effort was associated with longer response times, which were conceivably required for additional mental computation. Binaural processing may thus be more effortful in daily life for children who use bilateral CIs.

### Pupillary Responses Change with Normal Development

RT and pupil diameter decreased with time-in-sound (chronological age) in NH listeners, but not CI users (Figs. 8c and 9c, respectively). A similar result has been found at many different stages of cognitive processing and may reflect normal development of cortical connections supporting the formation of cognitive networks [118,119]. Auditory cortical responses continue to mature until approximately age 20 [93]. Children with NH or unilateral CIs show a large and broad positive P1/P2 peak in their cortical evoked waveforms for the first 7 years of time-in-sound [7,83]. As children reach 12 years of hearing experience, a smaller negative N1 peak bifurcates the positive peak into two separate P1 and P2 components with the development of thalomocortical and cortico-cortical connections in superficial layers of the auditory cortex [93,94]. The polyphasic waveform P1-N1-P2-N2 becomes clearly present in all listeners as listening experience increases beyond 12 years [7]. Notwithstanding that normal cortical responses emerge with CI use, an abnormally large P2 amplitude persists, which may signify increased attentional demands and/or multisensory integration [91,92,94] and perhaps explain why neither RT nor PCPD decreased with longer durations of time-in-sound in CI children. While CI use drives normal developmental changes at the level of the cortex, processing and attempting to fuse spectrally degraded input still requires additional mental resources. Longer periods of auditory experience cannot overcome such device limitations. Analogous compensatory mechanisms are evident during speech and music perception [95,96].

### Bilateral CIs Promote Interaction in the Auditory Brainstem

Physiological processing of bilateral input in the auditory brainstem is necessary but not sufficient for perceptual integration or fusion. As shown in Fig. 7a, large asymmetries in EABR wave eV latencies were associated with very poor binaural fusion. The deficiencies in myelination, neural conduction, synaptic function, and synchronous activity associated with mismatched EABR latencies [2,6] may limit the auditory brainstem’s ability to code and transmit a fused image to higher levels. Latency mismatches may be due to prolonged unilateral stimulation, inconsistent bilateral CI use, or an abnormality on one side (e.g., hypoplastic nerve) in the case of very large mismatches. While CI users do not appear to integrate binaural information perceptually like their NH peers (Fig. 3), data from our laboratory provide evidence that matched bilateral stimulation promotes interaction at the level of the auditory brainstem [4]. The binaural difference response was visible in all children with bilateral CIs who were tested when stimulation was delivered to the same apical electrodes. Clear peaks in the binaural difference response were present due to reductions in amplitude evoked by bilateral stimulation relative to the sum of unilaterally evoked responses. This response may be indicative of inhibitory activity associated with binaural processing [42–44]. The finding that binaural input interacts at the level of the brainstem, but that child bilateral CI users are unable to achieve binaural fusion is consistent with Aharonson and Furst’s model [117]. Binaural processing in the brainstem is clearly insufficient for the perception of a single auditory image, but is necessary to some degree to code changes in interaural timing and level differences.

Integration at higher centers in the auditory system is important for the perception of one fused auditory image. Fiedler and colleagues found that dichotically presented pure tones were both perceived as fused and integrated cortically, as indicated by the absence of a mismatch negativity waveform [46]. Despite normal cortical development promoted by CI use [5,6,94], deviations from normal remain [7,120]. Cortical responses would need to be evoked in CI users during participation in a binaural fusion task in order to determine whether abnormal cortical integration is in some way underlying observed shortcomings in perceptual integration. Alternatively, integration similar to NH listeners at the level of the cortex would suggest that, while CI stimulation promotes physiological integration, fusion is limited more by current speech processing schemes. If so, future CI devices should provide additional information, particularly regarding binaural timing cues, to promote binaural fusion.

## Summary and Conclusion

Children using bilateral cochlear implants perceive a single fused input from their two devices best when differences in interaural level are present. Binaural fusion is poor when interaural level cues are absent and further impaired when large asymmetries exist in the bilateral brainstem pathways. Better binaural fusion was associated with longer acoustic experience prior to cochlear implantation, reflecting the importance of acoustic hearing during auditory development. Reduced binaural fusion comes at a cost of increased listening effort as measured by reaction time and pupil diameter. Although benefits of bilateral CIs can be achieved without binaural fusion, efforts to promote this normal aspect of binaural hearing could further ease listening and improve hearing for children using these devices.
