# Correction: Binaural Fusion and Listening Effort in Children Who Use Bilateral Cochlear Implants: A Psychoacoustic and Pupillometric Study

**DOI:** 10.1371/journal.pone.0141945

**Published:** 2015-10-30

**Authors:** Morrison M. Steel, Blake C. Papsin, Karen A. Gordon

In Fig 4, there is an error with the legends for Normal Hearing and Cochlear Implant. Please view the correct Fig 4 here.

**Fig 4:**
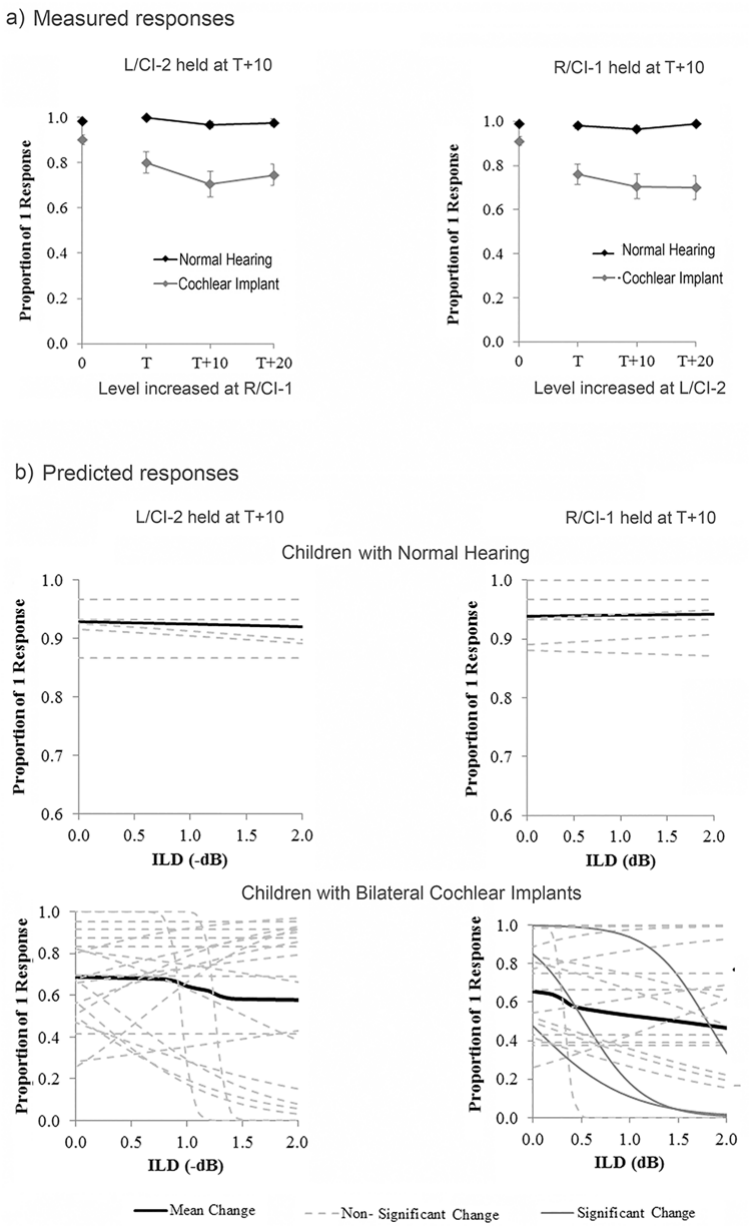
Fusion with interaural level differences. a) Group performance for conditions containing ILDs (ITD = 0 ms). Biphasic pulses were delivered from electrode 20 in the CI group (n = 25). CI listeners consistently perceived one image when there were level differences, albeit less frequently than NH peers (n = 24; p < 0.0001). b) Binaural fusion was predicted as a function of ILD for individual normal hearing children and CI users with logistic regression. None of the slopes were significant in the normal hearing children as shown by the dashed lines (p > 0.05). For CI users, the majority of curves tend to decrease as a function of increasing ILD. Significant slopes (n = 3) are represented by dark grey solid lines.
